# Benefits of semi-outdoor environments near classroom for restoring students’ cognitive performance: role of exposure duration

**DOI:** 10.3389/fpsyg.2026.1769577

**Published:** 2026-05-06

**Authors:** Cuina Zhang, Jiaxin Cao, Jiayun Wu, Yuting Cai, Yatong Wang, Yusha Wei

**Affiliations:** School of Engineering, Shantou University, Shantou, China

**Keywords:** attention restoration, cognitive performance, exposure duration, restorative benefits, semi-outdoor environments near classrooms

## Abstract

Although the restorative effects of classroom environments are well-documented, the mechanisms underlying semi-outdoor environments near classrooms (SENC), particularly with respect to exposure duration, remain unclear. This study examined the cognitive/neural effects of SENC using the Classroom Veranda Wandering Test (CVWT) and electroencephalograph (EEG). Seventy participants were randomly assigned to either a 5-min (*n* = 35) or 10-min (*n* = 35) exposure group in a between-subjects design, and cognitive performance was assessed before and after exposure in a within-subjects design. The results showed that SENC significantly improved cognitive performance, as indicated by reduced reaction times in the Spatial Memory Span Test (SMST). Mechanistically, SENC exposure was associated with increased *α*, *β*, and *θ* wave activity during exposure (*p* < 0.01) and further elevations in β and θ waves during the posttest (*p* < 0.05), indicating enhanced attention and cognitive control. Although both exposure durations facilitated restoration, the 10 min condition produced significantly greater benefits (*p* < 0.05). These findings provide empirical evidence for optimizing break-time management and integrating SENC as cognitive restoration spaces in campus design to enhance learning efficacy.

## 1 Introduction

Since the introduction of Attention Restoration Theory (ART; Kaplan and Kaplan, 1989) and Stress Restoration Theory (SRT; Ulrich et al., 1991), a large number of scholars have paid attention to research on restorative environments (Shibata and Suzuki, 2002; Tanner, 2009; Barrett et al., 2015; Lyu et al., 2022; Ko et al., 2023; Rhee et al., 2023). Several studies have demonstrated that restorative environments not only relieve stress, but also improve cognitive performance (Li and Sullivan, 2016; Korpela and Ratcliffe, 2021; Dzhambov et al., 2023; Wu et al., 2025). Systematic reviews have provided an evidence base for this relationship, suggesting that exposure to natural environments is associated with improved cognitive performance such as attention and executive function (Ohly et al., 2016). This foundation has been further deepened and expanded in recent research. Rhee et al. (2023) indicated that exposure to natural environments is conducive to improving cognitive performance and attention, while more comprehensive studies by Berman et al. (2019) and Yin et al. (2018) further confirmed that natural exposure can effectively enhance higher cognitive processes such as working memory and cognitive flexibility. At the same time, the restorative effects of learning spaces and the improvements in cognitive ability have led to increased research attention. Several studies have shown that plants and landscapes in learning spaces such as classrooms and schoolyards also play restorative roles (Rhee et al., 2023; Cosquer et al., 2025). Vásquez NG believe that the view around classroom windows is important for children, who tend to prefer natural views (Vásquez et al., 2019). A comprehensive study on campus common spaces further confirms that their characteristics, including architectural environment, landscape environment, and activity facilities, can significantly promote the psychological restoration of college students (Guo et al., 2023). Wu et al. (2025) found that restorative campus outdoor spaces, evaluated using street-view imagery and deep learning methods, were associated with reduced stress levels and improved mental well-being among students. Similarly, Cosquer et al. (2025) demonstrated that students who perceive their schoolyard as more natural report higher levels of psychological restoration and well-being. The visual landscape perceivable within the classroom helps students recover from stress (Peters and D'Penna, 2020). This landscape may consist of green spaces visible through the classroom windows (Li and Sullivan, 2016; Vásquez et al., 2019), or greenery located inside the classroom (Han, 2009; Rhee et al., 2023). The visual landscape perceivable within the classroom helps students recover from stress (Peters and D'Penna, 2020). This landscape may consist of green spaces visible through the classroom windows (Li and Sullivan, 2016; Vásquez et al., 2019), or greenery located inside the classroom (Han, 2009; Rhee et al., 2023). The feasibility of detecting stress within brief time windows (Pei et al., 2024) suggests that even transient exposure to semi-outdoor learning spaces may yield measurable physiological recovery. Restorative learning spaces also contribute to enhancing students’ cognitive abilities. Matsuoka (2010) found a consistent and systematic positive correlation between natural exposure and student performance. Semi-outdoor environments for work and study likewise play a role in cognitive recovery (Lyu et al., 2022). However, although research has confirmed the overall restorative effects of campus common spaces (Guo et al., 2023), the restorative potential of semi-outdoor environments near classrooms (SENC), such as classroom corridors and open floors typically adjacent to courtyards or green spaces, remains underexplored.

Unlike conventional restorative environments (e.g., forests and parks) or strictly indoor biophilic elements (e.g., indoor greenery), SENC represents an architectural-psychological hybrid characterized by the following features. First, from the perspective of architectural space typology, SENC falls under the category of “gray space,” which refers to the transitional zone between black space defined as a completely enclosed interior, and white space, defined as a completely open exterior. Examples include classroom corridors and pilotis levels that often feature views of natural landscapes or courtyards and are widely found in hot or rainy regions. These spaces are semi-enclosed, typically featuring coverings such as roofs, overhanging eaves, while the enclosure interfaces are open or translucent, such as railings, large glass windows, or grilles. Second, from a psychological mechanism perspective, SENC may possess the potential for “micro-restoration,” allowing students to temporarily escape from enclosed, high cognitive load environments during breaks, relieve mental fatigue, and achieve psychological relaxation. SENC is therefore a special space with specific spatial attributes and psychological functions; however, the mechanisms underlying its cognitive recovery effects have not been fully explored.

Electroencephalography (EEG) is an effective means of measuring changes in human psychophysiological states (Henriques and Davidson, 1991), and its time-and frequency-domain characteristics are often used to study environmental stress responses (Gandhi and Jaliya, 2024). Consequently, EEG has become a reliable method for exploring environmental quality through physiological measurements (Roe et al., 2013).

Regarding specific frequency bands, increased *α* band power is widely considered a sign of mental relaxation (Klimesch, 1999; Fachner et al., 2013). It has been extensively used to measure psychophysiological responses to natural environments, such as the perceived restorativeness of vegetation (Chang et al., 2008) and forest plant environments (Chiang et al., 2017). Conversely, a decrease in α band power is often observed when restorative elements are reduced (Rhee et al., 2023), and α oscillations have been found to be negatively correlated with stress, particularly in the prefrontal, parietal, and occipital regions (Jacobs and Friedman, 2004; Kaur and Singh, 2015; Al-shargie et al., 2018).

The role of *β* band activity in restorative environments is more ambiguous. While it is often examined alongside α activity (Rhee et al., 2023) and has been linked to dynamic plant interventions (Xu et al., 2025), its directional response remains debated. Some studies suggest that β activity increases in restorative environments (Chang et al., 2008), whereas others propose that a decrease indicates greater comfort and relaxation (Li et al., 2021). However, the β band is also strongly associated with attention and cognitive control (Wróbel, 2000). An increase in EEG β activity is often associated with positive attention (Fernández et al., 1995) and can reflect increases in selective visual attention (Rhee et al., 2023). For instance, Laumann et al. (2003) found that after exposure to a restorative environment, subjects showed increased β activity during attention-oriented tasks. This suggests that β-band oscillations in the prefrontal and parietal regions may reflect selective visual attention (Sabatinelli et al., 2014), leading to the hypothesis that stronger *β* oscillations during task execution indicate heightened attention. Therefore, whether β activity should increase or decrease in a relaxed environment remains an open question, particularly when distinguishing between stress reduction and cognitive engagement.

In contrast, *θ* band activity increases when the brain is in a relaxed, subconscious state. Participants exposed to restorative environments exhibit stronger θ band oscillations (Zhang et al., 2023). The brain shows significantly stronger functional connectivity in the θ band in these settings, and stronger θ oscillations in parietal regions suggest that the brain is physiologically prepared for better attentional readiness (Zhang et al., 2023). Generally, θ bands are associated with cognitive control processes such as conflict monitoring and working memory updating (Klimesch, 1999; Karakaş, 2020). Spontaneous θ oscillations confer operational readiness to the attentional system (VanRullen, 2016) and support executive control (Klimesch, 1999). As memory span increases, θ band power also increases (Onton et al., 2005), and it is generally accepted that increased θ oscillations indicate sufficient preparation for attention.

Despite extensive research, due to significant differences in variables, methods and environmental characteristics (Bringslimark et al., 2009), results regarding EEG and restorative effects remain inconsistent. Specifically, the relationship between EEG band changes, particularly the complex manifestations of *β* and *θ* bands during the exposure phase versus the task execution phase, and improvements in cognitive function requires further clarification.

In many restorative environment studies, exposure duration has mainly been around 3 min (Jeon et al., 2021; Latini et al., 2023), 5 min (Ko et al., 2020; Hsieh et al., 2023), or 10 min (Vásquez et al., 2019). However, some studies have examined durations of 1 min or even several tens of seconds (Abd-Alhamid et al., 2020; Li et al., 2021; Ghosh et al., 2022), as well as approximately 20 min (Lyu et al., 2022) and 60 min (He et al., 2023). Whether different exposure durations influence research outcomes remains unclear. Is there a difference in restorative effects across varying exposure durations? There is limited research addressing these issues. Gonçalves et al. (2023) pointed out that most studies have not assessed the duration required for exposure to meaningfully influence the variables.

This gap is particularly critical in educational settings. While recent policy reforms in some regions of China have extended break times to 15 min (Xinhua Net, 2025), durations such as 5 or 10 min are currently policy-driven and lack empirical support. Therefore, evidence-based guidelines for optimizing exposure duration are urgently needed.

To bridge these gaps, this study integrates multi-band EEG analysis with the temporal variable within the specific context of semi-outdoor environments near classrooms (SENC). Specifically, we aim to address the following research questions:

1. Can brief exposure to SENC effectively induce cognitive recovery, and does this restorative benefit vary with exposure duration?
2. How do *α*, β, and θ EEG bands change during the exposure phase (CVWT) to reflect neural relaxation, and how does exposure duration modulate these physiological responses?
3. How does SENC-induced attention restoration manifest in EEG activity during subsequent task execution, and do different exposure durations lead to significant differences in attention and cognitive control?

By elucidating the impact of exposure duration on restorative neurodynamics within SENC, this study deepens understanding of the mechanisms underlying micro-restoration. These findings provide a scientific basis for optimizing campus design and break-time management and offer empirical support for creating learning environments that effectively enhance student outcomes.

## 2 Methods

### 2.1 Study area

**Figure 1:**
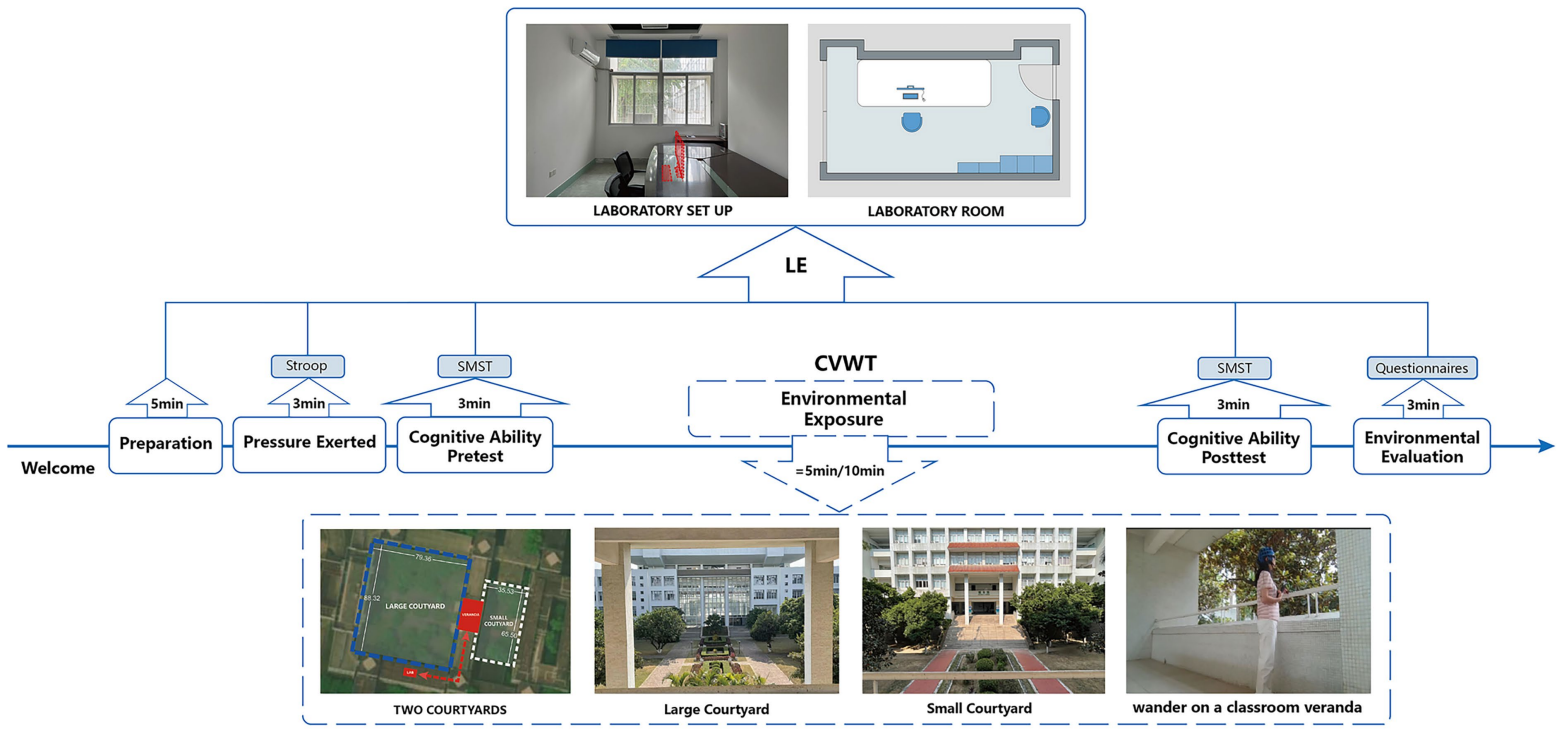
The location and procedure of experimental.

A Laboratory Experiment (LE) and a Classroom Veranda Wandering Test (CVWT) were conducted in this study. The LE was conducted in a Laboratory with internal dimensions of 6.66 × 3.24 × 4.00 m (height) in which there was a computer (HP Zbook, I9-12900HK/32GB DDRS:16G*2/1TBSSD/NVIDIA RTXA2000 8 GB, 16-inch, Kylin V10) at the School of Engineering, Shantou University, and the CVWT was conducted in a classroom veranda with two courtyards near the laboratory (Figure 1).

### 2.2 Procedure

There were 6 steps in this study’s experiment which lasted nearly 30 min including 5 steps in the LE and 1 step in the CVWT (Figure 1):

- Step1 (LE), Preparation (5 min): Participants wearing electroencephalography (EEG) equipment were required to complete a short demographic questionnaire and undergo a 3-min eyes-closed rest period to achieve psychological adjustment which has been shown to be sufficient for environmental immersion and psychological stabilization (Teplan et al., 2014).
- Step2 (LE), Pressure Exerted (3 min): The Stroop task which were conducted using a the computer in the laboratory was employed to induce psychological stress (MacLeod, 1992).
- Step3 (LE), Cognitive Ability Pretest (3 min): The Spatial Memory Span Test (SMST), used to measure cognitive performance (MacLeod, 1992), was administered for the first time as pretest before the CVWT.
- Step4 (CVWT), Environmental Exposure (5 min/10 min): Participants were randomly divided into two groups and wandered freely on a classroom veranda for 5 or 10 min.
- Step5 (LE), Cognitive Ability Posttest (3 min): SMST was completed second time as posttest after CVWT.
- Step6 (LE), Environmental Evaluation (3 min): A questionnaire was conducted including spatial preference evaluation by 7-points Likert scale and other restorative benefits evaluations to obtain subjective reactions from participants.

The participants in steps 1–6 above were all wearing electroencephalograph (EEG).

### 2.3 Participants

An independent-measures design was employed to compare the 5-min and 10-min intervention groups. A total of 70 participants (33 males, 37 females) were recruited from Shantou University. *A priori* power analysis using G*Power 21 determined that *N* = 70 would provide 80% power (1-*β* = 0.80) to detect practically significant effect sizes 38, a figure that also satisfies the central limit theorem for normality (Bagot et al., 2015). Statistical analyses included paired-samples t-tests for within-group pre-post comparisons and independent-samples t-tests for between-group comparisons. Inclusion criteria required psychological health, no history of neurological disorders, and normal or corrected-to-normal vision. Participants were instructed to abstain from alcohol and non-medical drugs for 24 h, from caffeine and strenuous activity for 12 h, and to have slept at least 8 h the night before. They also had to be free from colds for the past week. Compensation of 50 RMB was provided upon study completion. This study was approved by the Shantou University Ethics Review Committee for Human Related Science and Technology Research (STU20230613), and written informed consent was obtained from all individual participants prior to data collection.

## 3 Measures and analysis

### 3.1 Stroop task

The Stroop color-word interference test, first proposed by psychologist John Ridley Stroop in 1935, is a classic paradigm for studying cognitive conflict (Stroop, 1935). The experiment employs color words (e.g., “red,” “green”) printed in incongruent ink colors (e.g., the word “red” written in green ink), requiring participants to ignore the word meaning and rapidly name the ink color. This conflict between semantic and perceptual information induces strong cognitive interference, resulting in prolonged reaction times and increased error rates, thereby effectively generating psychological stress. In this study, the test served as a stress induction tool: standardized stimulus cards were presented to enable participants to accumulate cognitive load through sustained conflict tasks, simulating high-pressure states. This approach provides a controlled experimental foundation for subsequently examining the stress-alleviating effects of restorative environments.

### 3.2 Spatial memory span test

These tests are used to assess a person’s short-term memory, attention, and the ability to manipulate information in their mind. Performance on memory span tests can provide insights into cognitive abilities and may be used in clinical assessments, educational settings, or research studies.

The capacity of short-term memory is known as memory span, with the capacity of short-term memory being approximately 7 ± 2 information chunks. The research on memory span was first conducted by Jacobs (1887) based on Ebbinghaus’s series of recall after modifications. Spatial Memory Span Test (SMST) refers to the length of a series of spatial positions after stimuli are presented in fixed order, participants are shown a sequence of spatial locations or patterns and are asked to reproduce the sequence in the same order or in reverse order. SMST has important significance in practice and can be used as an indicator for professional ability assessment. In this research the retrieval of these spatial positions is consistent with their original presentation.

### 3.3 Standardization of the Classroom Veranda Wandering Test

During the Classroom Veranda Wandering Test (CVWT), participants were instructed to wander freely within the SENC. To ensure the validity of environmental exposure, participants were prohibited from using mobile phones, engaging in conversation, or being disturbed by other activities. The experiment was conducted under typical fair-weather conditions, ranging from sunny to partly cloudy, with natural daylight, while avoiding precipitation or extreme weather. Although participants’ behaviors were standardized, the experimental setting also ensured a stable testing environment. Specifically, the study location, situated within a campus courtyard adjacent to the teaching building, was naturally shielded from external traffic and sudden noise. Furthermore, the procedure was supervised by researchers to maintain order, thereby minimizing potential auditory disturbances during data collection.

### 3.4 Psychophysiological restorativeness

Psychophysiological measures such as EEG are widely recognized as effective indicators of changes in human psychophysiological states (Henriques and Davidson, 1991). Previous studies have employed EEG to assess psychophysiological restorativeness in environmental contexts (Chiang et al., 2017; Chen et al., 2020). Accordingly, EEG was selected as the measure of psychophysiological restorativeness in this study, with a focus on stress reduction, relaxation, and attention. A portable wearable electroencephalography system (Kingfar International Inc., China; BitBrain, 256 Hz) with a common mode rejection ratio (CMRR) of −115 dB and based on 10–20 electrode system was used for data acquisition. To ensure signal quality and capture specific neural oscillations, raw EEG data were preprocessed using a Butterworth bandpass filter set between 0.1 Hz and 49 Hz. This procedure removed DC drift and high-frequency artifacts while preserving the target frequency bands: theta (*θ*, 4–8 Hz), alpha (*α*, 8–12 Hz), and beta (*β*, 12–30 Hz). Specifically, electrodes FPZ, FZ, F3, and F4 located in the frontal lobe, which is primarily responsible for logic, memory, and higher-order cognitive tasks, were the primary focus during the Pretest and Posttest phases. Electrodes P3 and P4 located in the parietal lobe, associated with spatial attention and sensory integration, were analyzed during the SENC exposure and Posttest phases. Electrodes O1 and O2 located in the occipital lobe, related to visual processing, were monitored during the SENC exposure phase. The data were processed using the design module of ErgoLAB 3.0 (Kingfar International Inc.).

### 3.5 Data analysis

EEG data were processed using the ErgoLAB EEG analysis module, including high- and low-pass filtering (0.1–49 Hz) and frequency domain index extraction. In this study, the analyzed frequency bands included θ (4–8 Hz), α (8–12 Hz), and β (12–30 Hz; Canolty and Knight, 2010). The power spectral density (PSD) of each band was compared across different exposure duration groups and experimental stages.

We compared the *α* band changes during pretest and exposure stage to test whether Stroop compression was successful. The changes in the average correct reaction time and average reaction time during the pretest and posttest of SMST were examined to determine whether the cognitive ability of the subjects had improved. Analyze the changes in α band, *β* band and θ band during the CVWT stage compared to the pretest stage to test the relaxation effect of the SENC, and analyze the changes in β band and θ band during the CVWT stage compared to the posttest stage to test the changes in participants’ attention. The EEG-β band and EEG-θ band are closely related to attention (Behrmann et al., 2004; Sabatinelli et al., 2014; VanRullen, 2016), and we can assume that during the cognitive task execution phase, the enhancement of β band and θ band oscillations indicates an increase in sample attention.

Research has shown that *α* band is more pronounced in the occipital and parietal regions, with the parietal lobe region primarily associated with human attention (Behrmann et al., 2004). However, when working memory tasks or maintaining attention are required, the activity of *θ* band in the frontal region may increase. *β* band reflect the brain activity of actively thinking, paying attention, or solving specific problems, and are more pronounced in the frontal and central regions. Based on the above analysis, to further analyze the relationship between various EEG changes and the perceived changes of the subjects, we also focused on analyzing EEG changes in the occipital, parietal, frontal, and frontal regions. For the convenience of analysis, we combined the power of channels O1 and O2 in the pillow area to handle the common reflection of pillow area power. This method is also used in other areas.

The above experimental analysis used paired t-test, and we also compared the differences in the above results between the 10-min group and the 5Mins group using t-test. Statistical analyses were performed using IBM SPSS v. 24.

## 4 Results

### 4.1 Statistical tests and baseline homogeneity

#### 4.1.1 Test of normality

Normality of the sample data was assessed using the Jarque-Bera test. Given the strict requirements of normality tests, data were considered acceptably normal if the absolute value of skewness was less than 3 and kurtosis was less than 10 (Cromwell et al., 1994). For datasets that did not initially meet these criteria (including *α* (Preparation), βF (Pretest), β (Pretest), and β (Posttest)), outliers were identified and removed through box plot analysis. This procedure yielded distributions that satisfied the normality assumptions (skewness: −0.038–2.662; kurtosis: −0.078–9.125).

#### 4.1.2 Baseline characteristics comparison

**Figure 2:**
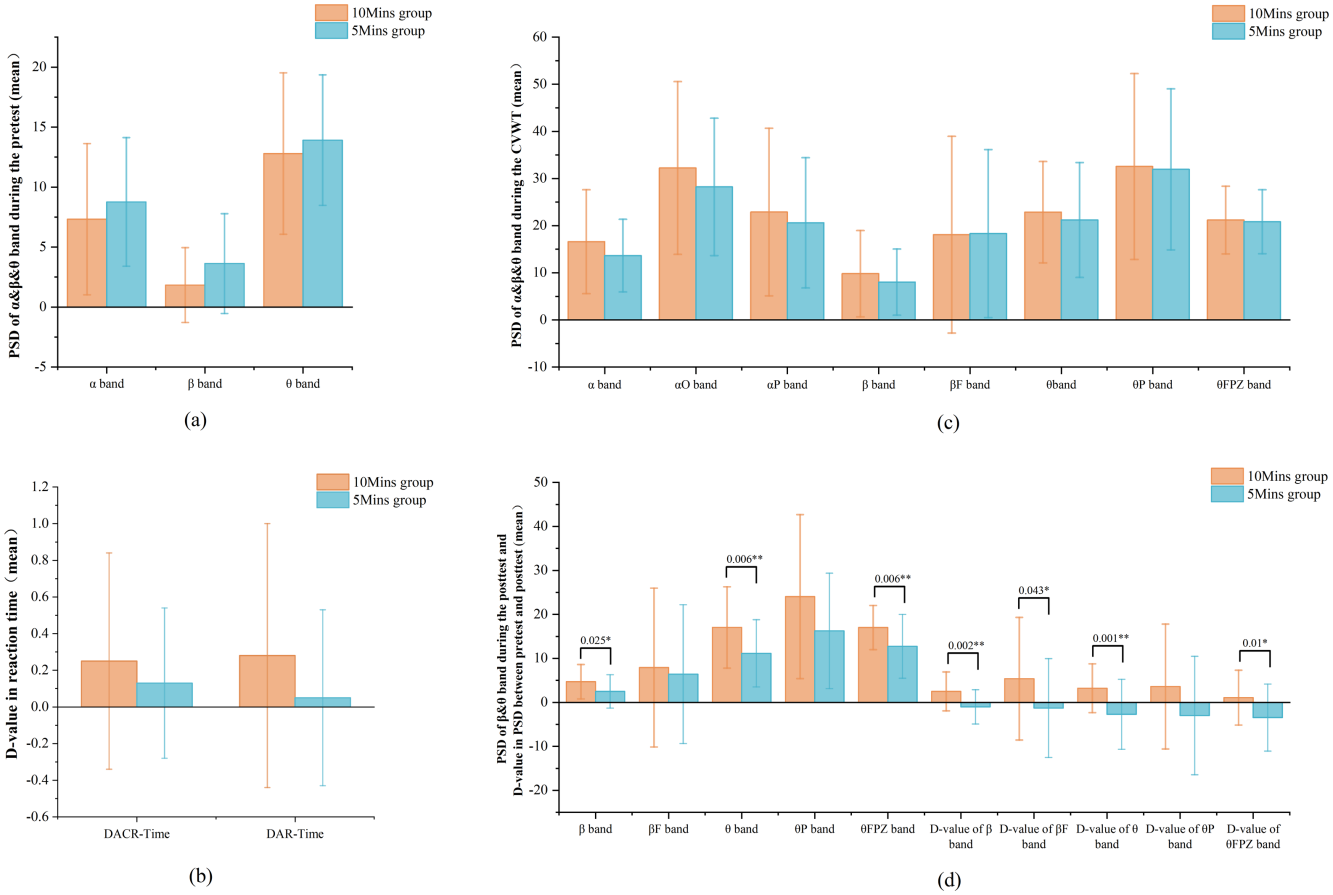
*T*-test of the difference between the 10Mins group and the 5Mins group. **(a)** The difference in PSD during the pretest stages. **(b)** The difference in D-value of reaction time between the pretest and posttest stages. **(c)** The difference in PSD during the CVWT stages. **(d)** The difference in PSD during the posttest and D-value in PSD between the pretest and posttest stages (*=significant at *p* < 0.05; **=significant at *p* < 0.01).

Baseline demographic characteristics, cognitive measures, and pretest neural activity were compared between the two groups using independent t-tests and chi-square tests. The chi-square test indicated no significant difference in gender distribution (χ^2^ = 0.516, *p* = 0.473; Table 1). Independent-samples t-tests demonstrated that the two groups did not differ significantly in any baseline cognitive measures, including accuracy (*t* = −1.174, *p* = 0.244, *d* = 0.281), average correct reaction time (*t* = 0.157, *p* = 0.876, *d* = 0.038), average reaction time (*t* = 0.458, *p* = 0.648, *d* = 0.112), or level threshold (*t* = −0.680, *p* = 0.499, *d* = 0.163; Table 2). Similarly, analysis of pretest PSD showed no significant between-group differences in the α (*t* = −1.016, *p* = 0.314, *d* = 0.246), β (*t* = −1.942, *p* = 0.057, *d* = 0.486), or θ (*t* = −0.753, *p* = 0.454, *d* = 0.183) bands (Figure 2a). Collectively, these results confirm successful randomization and baseline comparability.

**Table 1:** Chi-square test of gender distribution between the 10Mins group and the 5Mins group.

Gender	Time group (%)		Total (%)	χ^2^	*p*
	10Mins group	5Mins group			
Male	51.43	42.86	47.14	0.516	0.473
Female	48.57	57.14	52.86		

*Significant at *p* < 0.05; **Significant at *p* < 0.01.

**Table 2:** Independent-samples t-tests of pretest cognitive measures between the 10Mins group and the 5Mins group.

Cognitive measure	Time (mean ± standard deviation)		*t*	*p*	Cohen’s *d*
	10Mins group	5Mins group			
Pretest accuracy (%)	69.98 ± 8.36	72.18 ± 7.30	−1.174	0.244	0.281
Pretest average correct reaction time (s)	3.42 ± 0.88	3.39 ± 0.69	0.157	0.876	0.038
Pretest average reaction time (s)	3.97 ± 1.02	3.88 ± 0.69	0.458	0.648	0.112
Pretest level threshold	5.35 ± 0.90	5.51 ± 1.03	−0.680	0.499	0.163

*Significant at *p* < 0.05; **Significant at *p* < 0.01.

#### 4.1.3 Pressure verification

As shown in Table 3, the effectiveness of the pressure induction stage was confirmed by a significant decrease in EEG α band power from the Preparation stage to the Pretest stage (*t* = 3.427, *p* = 0.001, *d* = 0.428). This reduction was also significant in the 10Mins group (*t* = 2.258, *p* = 0.031, *d* = 0.406) and the 5Mins group (*t* = 2.544, *p* = 0.016, *d* = 0.443). The decrease in *α* oscillations is a well-established psychophysiological indicator of reduced mental relaxation and increased cognitive tension (Klimesch, 1999), thereby validating the effectiveness of the stress induction.

**Table 3:** Paired sample *T*-test of α band PSD between the preparation and pretest stages.

Samples	Pairing (mean ± standard deviation)		Difference	*t*	*p*	Cohen’s d
	α (Preparation)	α (Pretest)				
Total samples (*n* = 70)	10.38 ± 7.81	8.11 ± 5.96	2.27	3.427	0.001**	0.428
10Mins group (*n* = 35)	9.62 ± 7.67	7.51 ± 6.54	2.12	2.258	0.031*	0.406
5Mins group (*n* = 35)	11.08 ± 8.00	8.67 ± 5.41	2.41	2.544	0.016*	0.443

*Significant at *p* < 0.05; **Significant at *p* < 0.01; α (Preparation): The PSD of α oscillations during the Preparation; α (Pretest): The PSD of α oscillations during the Cognitive Ability Pretest.

### 4.2 Analysis of cognitive performance

#### 4.2.1 Comparison of pretest and posttest

**Figure 3:**
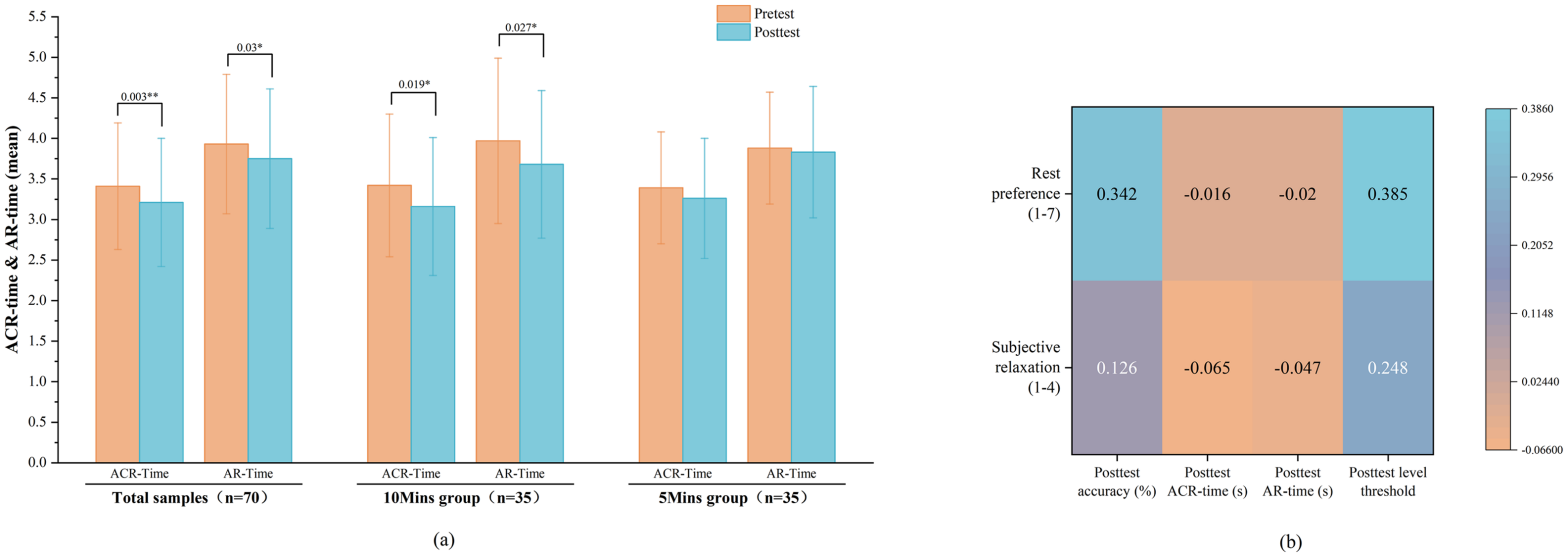
Analysis of cognitive performance and subjective response. **(a)** Paired sample T-test of ACR-time & AR-time between the pretest and posttest stages (*=significant at *p* < 0.05; **=significant at *p* < 0.01). **(b)** Correlation analysis between posttest cognitive responses and subjective preferences.

Consistent with findings that natural environments alleviate stress and enhance cognitive abilities (Ohly et al., 2016), this study compared the Spatial Memory Span Test (SMST) results before and after exposure to the semi-outdoor space. As shown in Figure 3a, paired-samples t-tests revealed that, compared with the pretest, the average correct reaction time (ACR-Time) and average reaction time (AR-Time) significantly decreased in the total sample (*t* = 3.107, *p* = 0.003, *d* = 0.380; *t* = 2.212, *p* = 0.030, *d* = 0.270) and in the 10Mins group (*t* = 2.467, *p* = 0.019, *d* = 0.429; *t* = 2.318, *p* = 0.027, *d* = 0.404), but not in the 5Mins group (*p* > 0.05).

#### 4.2.2 Posttest and subjective response

As shown in Figure 3b, Pearson correlation analysis between posttest cognitive responses and subjective preferences revealed a significant positive correlation between rest preference (1–7) and posttest accuracy (*r* = 0.342, *p* < 0.05), as well as posttest level threshold (*r* = 0.385, *p* < 0.05). This indicates that a higher preference for rest in SENC was associated with improved cognitive task performance in terms of accuracy and higher-level threshold among participants. However, rest preference showed no significant correlation with ACR-Time (*r* = −0.016, *p* > 0.05) or AR-Time (*r* = −0.020, *p* > 0.05. Subjective relaxation (1–4) was not significantly correlated with any posttest cognitive indicators, including accuracy (*r* = 0.126, *p* > 0.05), ACR-Time: *r* = −0.065, *p* > 0.05), AR-Time (*r* = −0.047, *p* > 0.05), or level threshold (*r* = 0.248, *p* > 0.05). In summary, these findings suggest that rest preference and subjective feelings of relaxation had limited influence on posttest cognitive responses.

#### 4.2.3 Difference test between two groups

Although the t-test revealed no significant differences between the 10Mins and 5Mins groups in the Difference of Average Correct Reaction Time (DACR-Time) and the Difference of Average Reaction Time (DAR-Time; *p* = 0.35, *d* = 0.225; *p* = 0.114, *d* = 0.383), as shown in Figure 2b, the 10Mins group exhibited greater mean improvements in reaction speed (DACR-Time: 0.25 ± 0.59 s; DAR-Time: 0.28 ± 0.72 s) than the 5 Mins group (DACR-Time: 0.13 ± 0.41 s; DAR-Time: 0.05 ± 0.48 s). These results suggest a potential trend toward greater cognitive benefit with longer exposure duration. This marginal effect warrants further investigation in future studies.

### 4.3 Analysis of relaxation

#### 4.3.1 Comparison oscillations in CVWT and pretest

**Figure 4:**
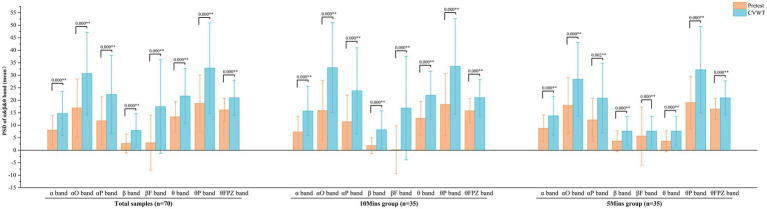
Paired sample *T*-test of the PSD between the pretest and CVWT stages (significant at *p* < 0.05; **=significant at *p* < 0.01).

Changes in EEG *α*, *β*, and *θ* band activity were measured during the Pretest and CVWT phases to assess participants’ relaxation levels (Chang et al., 2008; Zhang et al., 2023). A significant increase (*p* = 0.000**, *d* > 0.690) in the power spectral density (PSD) of all three bands was observed during the CVWT phase compared with the Pretest, indicating that participants entered a markedly relaxed state during SENC exposure (Figure 4).

#### 4.3.2 Comparison of relaxation between two groups

As shown in Figure 2c, a t-test comparison between the two groups revealed no significant differences in the PSD or change amplitude of the α, β, and θ bands during the CVWT phase. However, the 10Mins group consistently exhibited higher values for both measures than the 5Mins group, suggesting a non-significant trend toward greater physiological relaxation with longer exposure. In contrast to this physiological pattern, analysis of subjective relaxation ratings showed that the 5Mins group (2.03 ± 0.82) reported feeling more relaxed than the 10Mins group (1.60 ± 0.60; t = −2.486, *p* = 0.015, d = 0.594).

### 4.4 Analysis of attention effects

#### 4.4.1 Comparison β and θ band in pretest and posttest

**Figure 5:**
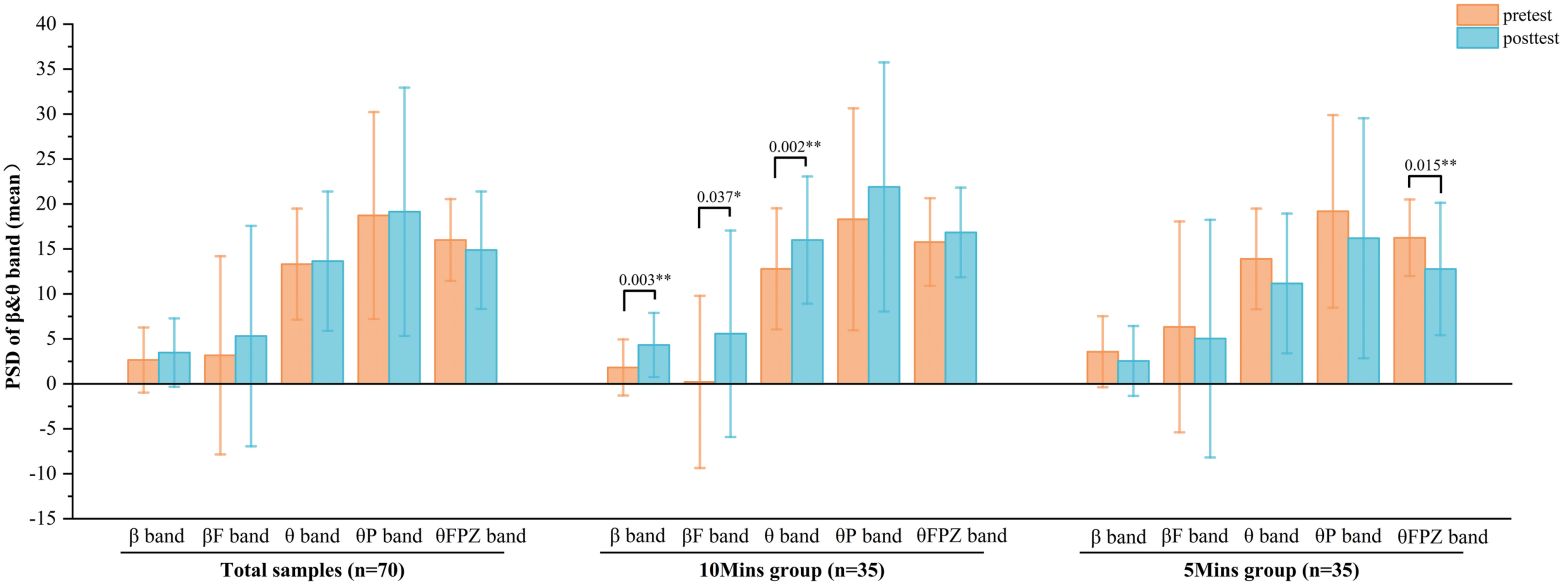
Paired sample *T*-test of the PSD between the pretest and posttest stages (significant at *p* < 0.05; **=significant at *p* < 0.01).

Reflecting active thinking, problem-solving, and attention, the β band is most prominent in the frontal and central brain regions. Both the β band (*t* = −3.186, *p* = 0.003, *d* = 0.563), βF band (*t* = −2.179, *p* = 0.037, *d* = 0.385) and θ band (*t* = −3.371, *p* = 0.002, *d* = 0.578) showed significant increases in the posttest for the 10Mins group refer to Figure 5, indicating that these participants achieved higher attentional efficiency. No significant increases in the β or θ bands were observed in the 5Mins group; instead, a decreasing trend was found in the θFPZ band (*t* = 2.571, *p* = 0.015, *d* = 0.454).

#### 4.4.2 Comparison attention effects in two groups

This inter-group difference was further confirmed by direct comparison. As shown in Figure 2d, the increase in the PSD of *β* (t = 3.281, *p* = 0.002, d = 0.841) and *θ* band (t = 3.525, *p* = 0.001, d = 0.868) from pretest to posttest was significantly greater in the 10Mins group than in the 5Mins group, a pattern also observed for the βF(t = 2.065, *p* = 0.043, d = 0.525), and θFPZ bands (t = 2.654, *p* = 0.01, d = 0.654). These results collectively indicate that the 10-min exposure produced a more pronounced improvement in attentional efficiency.

## 5 Discussion

### 5.1 Resting in SENC helps improve cognitive performance

This study demonstrates that SENC can significantly restore and enhance students’ cognitive performance. Participants who engaged in brief wandering on a classroom veranda with views of courtyards showed significant improvements in the SMST. Paired t-tests of the SMST results revealed statistically significant enhancements in memory ability, encompassing “accuracy,” “correct reaction time,” and “reaction time,” both in the total sample and in the 10Mins group. This finding aligns with prior research indicating that campus spaces featuring leisure landscapes also possess restorative benefits and contribute to enhanced student cognitive abilities (Li and Sullivan, 2016; Vásquez et al., 2019; Lyu et al., 2022; Rhee et al., 2023).

The SENC in this study, a type of semi-open architectural space integrating built and natural elements, demonstrated restorative effects comparable to those of more immersive natural environments. According to ART (Kaplan and Kaplan, 1989), exposure to nature or nature-like environments can restore directed attention. Our findings support this theory, indicating that brief rest periods in semi-outdoor courtyards allow students to restore attention and subsequently perform better on cognitive tasks. This effect is likely attributable to the inclusion of natural landscape features within the SENC, which, similar to other restorative environments, effectively facilitate attention restoration (Rhee et al., 2023).

Importantly, our findings the theoretical understanding of restorative environments, emphasizing that not only large green spaces or indoor plants but even “semi-open architectural spaces” adjacent to learning areas can facilitate cognitive restoration. Previous studies have highlighted the benefits of natural views, indoor vegetation, and landscaped campuses for students’ attention and stress levels (Rhee et al., 2023; Wu et al., 2025). We complement these studies by demonstrating experimentally that SENC improves cognitive performance. Theoretically, this study enriches the empirical foundation of the ART framework by incorporating the temporal dimension. Specifically, the findings suggest that SENC can function as an effective environmental intervention for cognitive restoration during short breaks, providing an evidence-based basis for optimizing restorative environments in educational settings.

### 5.2 SENC triggers relaxation and enhances attention through a two-stage neural process

This study provides an in-depth look at the dual-phase neural mechanisms of SENC in students’ cognitive recovery. During the CVWT, participants exhibited systematic enhancements in EEG oscillations of *α*, *β*, and *θ* waves across key brain regions including the parietal, occipital, frontal, and prefrontal lobes. These neural activity changes signify the neural restorative effects occurring during the CVWT phase. More importantly, this effect translated into attention restoration during cognitive task execution in the posttest phase, specifically manifested as sustained enhancements in β and θ waves during the posttest, directly confirming SENC’s attention-enhancing effect.

During the CVWT phase, the enhancement of α waves indicated that participants achieved a relaxed state. Significant α-wave enhancement in the parietal (spatial perception and attention allocation) and occipital (visual processing) lobes suggests their coordinated interaction during relaxation. This synergy may imply that within the SENC environment, students engage in relaxed exploration of their surroundings through visual perception of the natural landscape, which not only alleviates psychological stress but also promotes an optimal state of attentional readiness for subsequent tasks. These findings are consistent with previous research showing that increased α-wave activity typically reflects neural relaxation (Klimesch, 1999; Fachner et al., 2013).

The observed increase in β band power during the SENC exposure and the subsequent further elevation during the posttest highlights a complex interaction between environmental stimulation, neural arousal, and cognitive engagement. Traditionally, there is no consensus on whether beta waves increase or decrease during restorative environmental exposure. Some studies have shown that β activity increases in restorative environments (Chang et al., 2008), while others have suggested that a decrease in β activity indicates a higher level of comfort and relaxation (Li et al., 2021). In this study, the increase in β band during the SENC phase aligns with the previous perspective: it likely reflects mild cognitive arousal driven by the engagement with specific environmental features of the SENC, such as being in close proximity to landscaped courtyards, lawns, and trees. This environmental arousal serves as a “preparatory” mechanism for the subsequent cognitive task. As a result, during the posttest phase, participants exhibited significantly higher *β*-band power compared to the pretest, indicating enhanced attentional efficiency (Fernández et al., 1995; Laumann et al., 2003; Sabatinelli et al., 2014). This suggests that the SENC not only mitigated the mental fatigue accumulated during Pretest but also prepared the neural substrate for high-efficiency cognitive performance.

The observed *θ*-wave enhancement during SENC exposure signifies effective restoration of student attention, preparing them for subsequent cognitive experiments (Zhang et al., 2023). This finding aligns with prior evidence that interaction with restorative environments—such as natural settings—enhances θ oscillations (Behrmann et al., 2004). Given that θ oscillations are intrinsically linked to cognitive control (Klimesch, 1999) and their power scales positively with memory span (Onton et al., 2005), the sustained increase in *θ*-wave power observed during the posttest provides direct neurophysiological evidence of improved attentional engagement during task execution. By demonstrating this persistence from the exposure phase to active performance, our findings offer mechanistic insights into the previously debated “inconsistent effects of restorative environments on attention” (Bringslimark et al., 2009).

### 5.3 The 10Mins group achieved a higher level of attention

This study demonstrates the restorative effect of environmental exposure on students’ cognitive abilities, with its primary contribution being the clarification of the critical influence of exposure duration on the restoration process. By comparing two exposure durations, 5 and 10 min, the results showed that although both durations yielded cognitive restoration benefits, the 10-min exposure produced more pronounced restorative effects.

From a behavioral perspective, t-tests revealed no significant differences between the 5Mins and 10Mins groups in SMST. However, the 10Mins group demonstrated numerically superior performance across all indicators, including higher accuracy and faster response times (lower ACR-Time and AR-Time) compared to the 5Mins group, although these differences were not statistically significant. The larger mean improvements in reaction speed (0.25 ± 0.59 vs. 0.13 ± 0.41, 0.28 ± 0.72 vs. 0.05 ± 0.48) suggests a potential trend toward greater improvement in reaction speed with longer exposure. This pattern indicates a tendency toward greater improvement in accuracy and response speed for the 10Mins group during the posttest, reflecting a slight behavioral advantage. The subjective questionnaire indicated that the 5Mins group felt more relaxed (2.03 ± 0.82 VS. 1.60 ± 0.60) during the CVWT stage. The lower subjective relaxation reported by the 10Mins group is likely attributable to the prolonged discomfort associated with wearing the EEG cap, as the device’s tight fit and wet electrodes (requiring conductive paste between the scalp and electrodes) may have produced a sensation of pressure. This discomfort, amplified during the extended 10-min exposure, may have influenced subjective perceptions, as some participants reported that the EEG cap caused discomfort, which is caused discomfort, which is consistent with previous findings regarding device-induced discomfort during prolonged physiological monitoring (Hanzal et al., 2023). From a physiological perspective, however, EEG signals showed no significant differences between the 10Mins and 5Mins groups in terms of PSD of *α*, *β*, and θ waves during the CVWT phase. Notably, the 10Mins group exhibited higher mean PSD values for all three bands (α: 16.58 ± 11.03 vs. 13.63 ± 7.70; β: 9.80 ± 9.16 vs. 8.01 ± 7.02; θ: 22.85 ± 10.75 vs. 21.19 ± 12.19), suggesting that the 10Mins group tended to achieve greater neural relaxation, interest arousal, and attentional readiness at the physiological level.

Furthermore, in combination with the physiological indicators, comparison of the pretest and posttest results revealed that after the CVWT, participants exhibited higher β-wave power in the posttest. According to the t-test, the increase in the PSD of β waves in the 10Mins group was significantly higher than that in the 5Mins group, indicating that the 10Mins group demonstrated greater enhancement of attention, further confirming that the 10-min exposure exerted a stronger effect on students’ cognitive ability. Concurrently, during the posttest, the overall average θ-wave power and the θ-wave power in the frontal and parietal lobes were also significantly enhanced in the 10Mins group, reflecting superior attention restoration during task execution. These differences in physiological data robustly reinforce the conclusion that “exposure in the 10Mins group promoted stronger attention restoration.” In terms of theoretical value, this study adds an important “temporal dimension” to the framework of ART. While most restoration theories emphasize the role of environmental quality in cognitive restoration, the optimal duration of brief restorative breaks remains undefined. This study demonstrates the effectiveness of short-term exposure (5 and 10 min) in promoting relaxation, attention, and cognitive improvement, consistent with findings that even brief contact with nature can be beneficial (Zhang et al., 2023), while longer exposure may provide additional advantages (Maas, 2006).

Our findings, which indicate that 10 min of exposure is more effective than 5 min, align with the general trend proposed by Mollazadeh and Zhu (2021) that longer durations of natural exposure facilitate greater recovery. Although our study did not reach their recommended threshold of “>10 min,” it provides indirect support by clarifying the temporal distinctions within short exposure intervals (5–10 min), an area that has received limited systematic investigation (Gonçalves et al., 2023).

### 5.4 Limitations and future directions

This study has several limitations that should be addressed in future research. First, regarding exposure duration, we compared only two durations (5 and 10 min). Future research should examine a broader range of exposure durations (e.g., 5, 10, 15, or 20 min) to determine the optimal break duration and whether restorative effects plateau after a certain period. Additionally, incorporating control groups (e.g., indoor rest) would help isolate the specific effects of SENC from the general benefits of rest, thereby addressing the possibility that the observed effects may not be solely attributable to the semi-outdoor environment.

Second, the homogeneity of the sample limits the generalizability of the findings. All participants were students from a single university, and individual preferences for natural environments were not controlled, which may have influenced restorative responses. Future studies should include more diverse populations (e.g., students from different educational backgrounds and age groups) to enhance external validity. Moreover, the Stroop task used as a stress induction tool lacked independent validation of its effectiveness (e.g., subjective stress ratings). Relying solely on decreased α waves as a stress indicator may be insufficient; therefore, future research should combine physiological measures (e.g., α waves) with subjective stress assessments to confirm that the task effectively induced stress.

Third, although EEG provided valuable data, it captured only part of the complex restoration process. The CVWT procedure did not standardize ambient noise levels, which represents a potential confounding factor that may have interacted with SENC exposure to influence cognitive outcomes. Future studies should control for environmental variables (e.g., noise) during the CVWT to clarify the unique contribution of SENC. In addition, incorporating multimodal data (e.g., eye-tracking for attentional engagement and heart rate variability for autonomic responses) would provide a more comprehensive understanding of the mechanisms underlying the restorative effects of SENC.

## 6 Conclusion

This study demonstrates that resting in a SENC effectively promotes relaxation and enhances students’ cognitive performance. Notably, while both 5Mins group and 10Mins group exposures facilitated restoration, the 10Mins group condition elicited significantly stronger neural indicators of attentional engagement during subsequent cognitive tasks, alongside more pronounced improvements in behavioral performance. These findings provide empirical support for optimizing break-time management in educational settings, suggesting that extending break durations beyond standard short intervals can maximize cognitive restoration. Despite certain limitations, this study establishes an important foundation for integrating semi-outdoor environments into campus design to support student cognitive functioning.

## Data Availability

The original contributions presented in the study are included in the article/Supplementary material, further inquiries can be directed to the corresponding author.
