# Supplementary material for: Benefits of semi-outdoor environments near classroom for restoring students’ cognitive performance: role of exposure duration
Source: Front Psychol. 2026 May 6;17:1769577. doi: 10.3389/fpsyg.2026.1769577 (PMC13187519; doi:10.3389/fpsyg.2026.1769577)
Supplement: Supplementary file 1 [file Table_1.docx]

***Supplementary Material***

**Table S1.** Paired sample T-test of the PSD of α&β&θ band between the pretest and CVWT stages

| Samples | | Pairing (mean ± standard deviation) | | Difference | t | p |
| --- | --- | --- | --- | --- | --- | --- |
|  |  | Pretest | CVWT |  |  |  |
| Total samples (n=70) | α band | 8.04±5.85 | 14.75±8.86 | -6.7 | -7.425 | 0.000** |
|  | αO band | 16.91±11.61 | 30.71±16.49 | -13.81 | -7.873 | 0.000** |
|  | αP band | 11.78±9.61 | 22.32±15.62 | -10.54 | -5.630 | 0.000** |
|  | β band | 2.73±3.76 | 7.91±6.73 | -5.19 | -6.132 | 0.000** |
|  | βF band | 2.95±10.94 | 17.47±18.74 | -14.52 | -5.873 | 0.000** |
|  | θ band | 13.35±6.10 | 21.68±10.97 | -8.33 | -7.443 | 0.000** |
|  | θP band | 18.68±11.39 | 32.86±18.08 | -14.19 | -6.827 | 0.000** |
|  | θFPZ band | 16.12±4.56 | 21.00±6.99 | -4.88 | -6.003 | 0.000** |
| 10Mins group (n=35) | α band | 7.32±6.30 | 15.70±9.87 | -8.38 | -5.977 | 0.000** |
|  | αO band | 15.87±12.05 | 33.02±17.99 | -17.15 | -6.519 | 0.000** |
|  | αP band | 11.42±10.59 | 23.79±17.22 | -12.37 | -4.692 | 0.000** |
|  | β band | 1.83±3.12 | 8.20±7.53 | -6.36 | -4.521 | 0.000** |
|  | βF band | 0.22±9.58 | 16.87±20.66 | -16.65 | -4.324 | 0.000** |
|  | θ band | 12.79±6.73 | 21.99±9.60 | -9.20 | -6.804 | 0.000** |
|  | θP band | 18.30±12.34 | 33.53±19.10 | -15.23 | -5.386 | 0.000** |
|  | θFPZ band | 15.77±4.87 | 21.04±7.25 | -5.26 | -4.383 | 0.000** |
| 5Mins group (n=35) | α band | 8.76±5.36 | 13.79±7.75 | -5.03 | -4.638 | 0.000** |
|  | αO band | 17.94±11.24 | 28.40±14.76 | -10.46 | -4.733 | 0.000** |
|  | αP band | 12.14±8.66 | 20.85±13.93 | -8.71 | -3.274 | 0.002** |
|  | β band | 3.62±4.16 | 7.63±5.94 | -4.01 | -4.382 | 0.000** |
|  | βF band | 5.69±11.66 | 18.07±16.91 | -12.38 | -3.974 | 0.000** |
|  | θ band | 3.62±4.16 | 7.63±5.94 | -4.01 | -4.382 | 0.000** |
|  | θP band | 19.05±10.52 | 32.20±17.26 | -13.15 | -4.271 | 0.000** |
|  | θFPZ band | 16.48±4.27 | 20.96±6.82 | -4.49 | -4.052 | 0.000** |

Abbreviation: *=Significant at p<0.05; **=Significant at p<0.01
